# Preparation of Electrochemical Biosensor for Detection of Organophosphorus Pesticides

**DOI:** 10.1155/2014/303641

**Published:** 2014-12-24

**Authors:** Ashish Gothwal, Puneet Beniwal, Vikas Dhull, Vikas Hooda

**Affiliations:** ^1^Centre for Biotechnology, Maharshi Dayanand University, Rohtak 124001, India; ^2^Department of Bio- & Nanotechnology, Guru Jambeshwar University of Science & Technology, Hisar 125001, India

## Abstract

Polyvinyl chloride (PVC) can be used to develop reaction beaker which acts as electrochemical cell for the measurement of OP pesticides. Being chemically inert, corrosion resistant, and easy in molding to various shapes and size, PVC can be used for the immobilization of enzyme. Organophosphorus hydrolase was immobilized covalently onto the chemically activated inner surface of PVC beaker by using glutaraldehyde as a coupling agent. The carbon nanotubes paste working electrode was constructed for amperometric measurement at a potential of +0.8 V. The biosensor showed optimum response at pH 8.0 with incubation temperature of 40°C. *K*_*m*_ and *I*_max_ for substrate (methyl parathion) were 322.58 *µ*M and 1.1 *µ*A, respectively. Evaluation study showed a correlation of 0.985, which was in agreement with the standard method. The OPH biosensor lost 50% of its initial activity after its regular use for 25 times over a period of 50 days when stored in 0.1 M sodium phosphate buffer, pH 8.0 at 4°C. No interference was observed by interfering species.

## 1. Introduction 

Organophosphate (OP) compounds are used as pesticides and insecticides in agriculture and have found applications in military practices as chemical warfare agents. OP compounds contribute about 38% of the total pesticides used worldwide [1]. Commonly used OP pesticides include parathion, malathion, methyl parathion, chlorpyrifos, diazinon, dichlorvos, phosmet, and fenitrothion. According to World Health Organization reports, there are three million pesticide poisonings that occurred worldwide every year; most of them are OP-related, and 200,000 deaths occur due to either self-poisoning or occupational exposure [2]. Reports in the literature demonstrate that OP pesticides affect the nontarget organisms such as birds and fish, as well as humans through exposure to water resources, fruits, vegetables, and processed foods contaminated by OP pesticides. The OP pesticides irreversibly inhibit the activity of acetylcholine esterase, an enzyme essential for the proper functioning of the central nervous system in humans and insects, resulting in the accumulation of the neurotransmitter acetylcholine in the nerve cells and interfere with muscular responses and cause serious problems and sometimes fatal [3–5]. Analytical methods for determination of these toxic compounds are required to ensure that environment and human health will not be compromised by the usage of OP pesticides. There are many laboratory based analytical methods which are commonly used for the determination of OP pesticides. These include gas chromatography (GC), high-performance liquid chromatography (HPLC), capillary electrophoresis [6, 7], mass spectrometry [8], and thermospray-mass spectrometry [9]. Besides the sensitivity and accuracy of these methods, they are expensive and time-consuming and require pretreatment of the sample, as well as requiring highly trained persons to perform them, and are not suitable for in-field analysis [8, 10, 11].

The need for rapid and cost-effective analytical methods for field analysis of OP pesticides has developed many techniques, in which few of them are bioanalytical in nature. These techniques are based on the inhibition assay [12] and immunoassay [13]. Enzyme-linked immunosorbent assay (ELISA) is quite sensitive assay for OP pesticides but, like other immunoassays, can detect only single compound and require multiple time-consuming steps. Inhibition assays based on cholinesterase activity are also used for screening processes. But these methods are not well suited for process control monitoring assays where fast and repetitive analyses are required [14].

These problems can be solved by the use of biosensor technology which fulfils the demands for on-site monitoring and the rapid detection of neurotoxic agents [15]. Biosensors based on the acetylcholine esterase (AChE) inhibition have been widely used for the determination of OP pesticides [16]. The main drawback of inhibition-based biosensors involves multiple-step operation, such as time-consuming incubation and reactivation/regeneration steps [14, 17].

Organophosphorus hydrolase (OPH) can catalyze the hydrolysis of a large number of OP pesticides [18]. The enzymatic hydrolysis of OP pesticides involves a pH change, as well as the generation of chromophoric species [19]. OPH-based assays are direct and respond to OP pesticides as substrate rather than inhibitors in case of inhibition-based biosensors. Consequently, these assays can be reversible and require only target analyte [14]. The main problem associated with these methods involves the use of free enzyme which can be used once and also increases the cost of procedure. To overcome this problem, use of immobilization method for confining the enzyme molecules onto the different insoluble supports, this enhances the reusability of enzyme through immobilization.

Although OPH-based methods have several advantages such as selectivity for target analyte and show fast response, they are less sensitive and have low detection limits as compared to inhibition-based methods [20, 21].

These problems can be overcome by using the carbon nanotubes (CNTs) as transducer material in amperometric biosensors. CNT showed excellent electrocatalytic properties with larger surface area [22]. Deo et al., which significantly enhanced the amperometric signals generated from OP pesticides [23]. The OPH biosensors provide a simple, fast, accurate, and selective monitoring of OP pesticides, as well as being good for in-field applications and monitoring of detoxification processes [19].

In the present method, the OPH was extracted and purified from* Brevundimonas diminuta *cells and used for the development of electrochemical biosensor by covalent immobilization of enzyme on to polyvinyl chloride (PVC) surface rather than working electrode. This facilitates the opportunity to change the working electrode on deterioration, without wasting the immobilized enzyme which makes it economical and convenient. The use of carbon nanotubes for the construction of working electrode provides efficient charge transfer from one phase to another. The use of polyvinyl chloride (PVC) in bioanalytical techniques will open a new pathway in biosensor design and fabrication, diagnostic kits, and related biomedical instrumentation. 

## 2. Materials and Methods

### 2.1. Chemicals and Reagents

Sephadex G-100 was purchased from MP Biomedicals. Nutrient broth, tris-HCL, EDTA, lysozyme, sucrose, and sodium hydroxide were purchased from Himedia. Glutaraldehyde (grade 1.25%) was purchased from Sigma chemicals, and organophosphorus (OP) pesticide (methyl parathion) was purchased from Shri Ram Fertilizers & Chemicals. All other chemicals were of analytical reagent grade unless otherwise stated. Double distilled water (DDW) was used throughout the experiments. Silver wire and PVC (polyvinyl chloride) beaker were bought from local market.

### 2.2. Instruments and Equipment

Cyclic voltammetry (CV) measurements were performed on a potentiostat/galvanostat (PGSTAT12/30/302, Autolab) with a three-electrode system composed of a platinum (Pt) wire as an auxiliary electrode, an Ag/AgCl electrode as reference electrode, and carbon nanotubes paste based working electrode. UV spectrophotometer (Shimadzu Corporation, Japan), temperature controlled water bath (SunRon), digital pH meter (EUTECH), refrigerator (LG), microwave oven (LG), rotatory incubator shaker (HICON), magnetic stirrer (HICON), refrigerated centrifuge (SIGMA), and deep freezer (Voltas). The study of surface morphology was carried out with scanning electron microscope (SEM) (Model Joel JSM-6510, Japan) at Department of Chemistry, M. D. University, Rohtak, India.

### 2.3. Microorganism

The lyophilized sample of bacteria* Brevundimonas diminuta* (MTCC 3361) was purchased from IMTECH, Chandigarh.

### 2.4. Revival and Growth of Bacteria

Lyophilized sample of* B. diminuta* was mixed with 5 mL of nutrient broth (pH-8.0) in a test tube to make a bacterial suspension. The conical flask (250 mL) containing 100 mL nutrient broth (pH-8.0) was inoculated with bacterial suspension. The culture media were incubated in a rotatory incubator shaker at 200 rpm for 24 hours at 30°C. Growth of bacteria was checked by taking O.D. at 600 nm.

### 2.5. Extraction and Purification of OPH from* Brevundimonas diminuta*

After desirable growth, the cells were centrifuged at 8000 rpm for 10 minutes. The cell pellets was incubated with 0.75 M Sucrose, mixed in 7.5 mL of 50 mM Sodium Phosphate Buffer, pH 8 at 20°C for 10 minutes. The suspension war treated with lysozyme as described by Herzliger et al. 1984 [24]. the suspension was centrifuged at 10000 rpm for 5 minutes. The pallet was separated and re-suspended in 20 mL of 50 mM sodium phosphate buffer (pH 8) containing 50 mM NaCl at 4°C for one hour with constant steering [25]. After centrifugation at 10000 rpm for 5 minutes the supernatant was separated and treated as crud enzyme solution. The crude solution was concentrated using lyophilization machine. Purification of concentrated crude Organophosphate hydrolase was done using sephadex G-100 column (2.5 × 30 cm) as described by Liu et. al. 2004 [26].

### 2.6. Enzyme Assay and Protein Estimation

The 0.1 mL of purified Organophosphate hydrolase enzyme solution was mixed with 2.9 mL Sodium Phosphate Buffer (pH 8), 5 nM methyl parathion (0.1 mL) was added to the enzyme solution and incubated at room temperature for 10 minutes. The spectrophotometric reading of the assay was taken at 410 nm [27]. The total protein content of the purified Organophosphate hydrolase enzyme solution was measured by Lowery method [28].

(1) OP  pesticide+H2O→OPH4-nitophenol+diethyl  phosphateλ=410 nm

### 2.7. Covalent Immobilization of OPH onto Inner Surface of PVC Beaker

Covalent immobilization of organophosphorus hydrolase onto PVC beaker surface was done by using the method described by Hooda et al. [29]. Inner surface of PVC beaker was filled with 5 mL of enzyme solution and kept overnight at 4°C. The surface was washed with buffer to remove the unbound enzyme. The reaction beaker was stored at 4°C containing sodium phosphate buffer (50 mM, pH-8.0).

### 2.8. Scanning Electron Microscopy of PVC Surface

To confirm the immobilization of enzyme, the scanning electron microscopic (SEM) study of the surface of PVC beaker was carried out, before and after enzyme immobilization at Department of Chemistry, M. D. University, Rohtak, India.

### 2.9. Preparation of Carbon Nanotubes Paste Working Electrode

**Figure 1:**
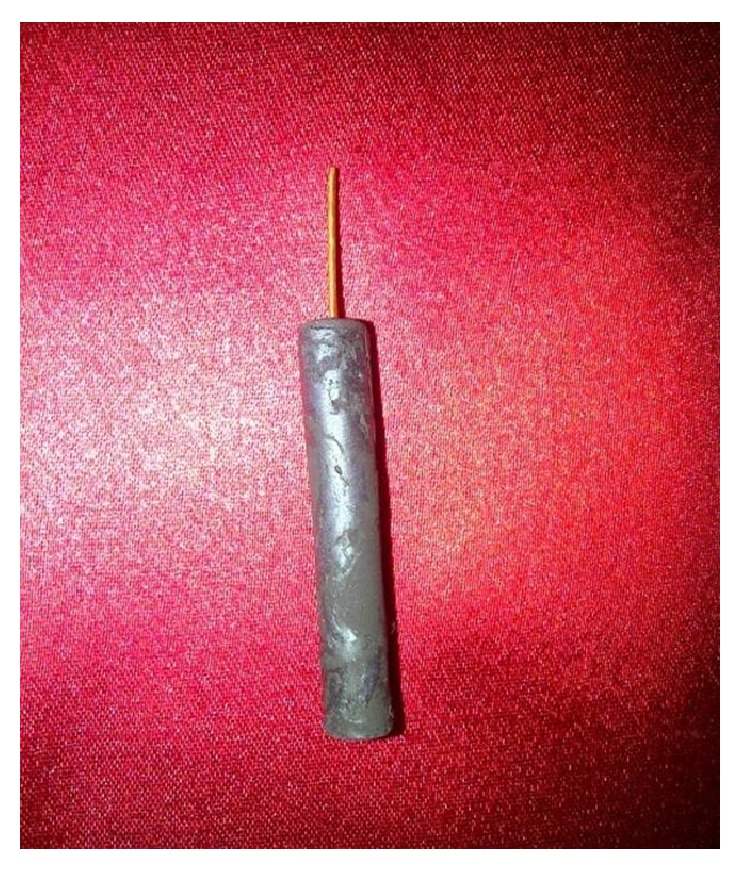
Carbon nanotubes paste working electrode.

Carbon nanotubes powder (1.0 g) and NH_4_Cl (0.2 mg) were mixed with paraffin oil in a ratio to obtain the consistency of paste [29]. This paste was filled in a plastic hollow tube (2 cm length and 4 mm diameter) with one closed end. Electrical contact was made by inserting a silver wire into the carbon nanotubes paste at one end (Figure 1). This formed the body of the working electrode. The surface of the electrode was washed with buffer and stored at 4°C when not in use.

### 2.10. Assembly of OPH Biosensor and Electroanalytical Measurements

**Figure 2:**
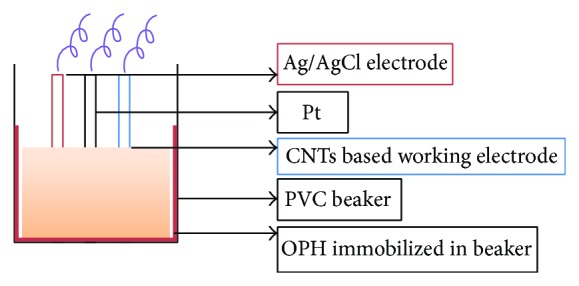
Basic assembly of OPH biosensor.

An amperometric OPH biosensor was developed by using enzyme immobilized PVC reaction beaker along with the three electrodes, that is, carbon nanotubes paste as working electrode, Ag/AgCl electrode as reference, and Pt wire as auxiliary electrode and being connected through potentiostat/electrochemical analyzer (Figure 2).

The electroanalytical measurements for OP pesticides were performed using a potentiostat/electrochemical analyzer. The cyclic voltammetric study of 4-nitrophenol was done in 50 mM sodium phosphate buffer (pH-8.0) in a reaction beaker cell at room temperature. In the cyclic voltammetry experiments, the scan rate was 50 mV/sec and the scan range was 0 V to +1.0 V.

### 2.11. Kinetic Properties of Immobilized OPH-Based Biosensor

The following kinetic properties of OPH biosensor were studied: optimum pH, incubation temperature, time of incubation, effect of substrate (methyl parathion) concentration, and calculation of *K*_*m*_ and *I*_max⁡_ from Line weaver - Burk plot between reciprocal of substrate concentration (1/[*S*]) and reciprocal of amount of the current (1/[*I*]).

### 2.12. Evaluation of the Present Method

The evaluation of present method was carried out by studying its linearity, minimum detection limit, analytical recovery, precision, and accuracy. The effect of interfering species on the response of biosensor was studied. The storage stability and reusability of present method were also determined.

#### 2.12.1. Linear Working Range and Minimum Detection Limit

The linearity and minimum detection limit were calculated by correlating the values with standard graph.

#### 2.12.2. Analytical Recovery

To determine the reliability of the method, different concentrations of methyl parathion (5 and 10 *μ*M) were added to the samples and the mean analytical recovery was determined by the present method.

#### 2.12.3. Precision

To study the reproducibility of the present method, the OP pesticide level was determined in the sample on the same day (within batch) and in the same sample after storage at 4°C for one week (between batch), coefficients of variation (CVs) were calculated for the present method.

#### 2.12.4. Accuracy

To evaluate the accuracy of present method, the levels of methyl parathion in 5 spiked water samples were determined by the present method and compared with those obtained by standard method.

#### 2.12.5. Reusability and Storage Stability of the Present Method

Before reuse of the OPH biosensor, it was washed by washing buffer (0.01 M phosphate buffer saline, pH-7.2 with 0.1% tween 20). The storage stability of the present biosensor was investigated over a period of two months, when reaction beaker was stored at 4°C. The response of present method was measured once in every 5 days.

#### 2.12.6. Effect of Interfering Species

The amperometric response was also checked in the presence of potential interfering species such as glucose, fructose, and sucrose as well as metal ions (Zn (II), Cu (II), Cd (II), Ni (II), and Pb (II)) each at a concentration of 2 *μ*M.

### 2.13. Application of Newly Developed OPH Biosensor

The water and food samples prone to be contaminated with OP pesticides determination were taken for study. Water samples which are used for the drinking purpose were used directly for the determination of OP pesticides. The food samples, that is, vegetables and fruits, (cauliflower, cabbage, grapes, and apple) were bought from local market. The food samples were washed with distilled water and chopped. 100 grams of each chopped sample was crushed in pestle mortar and homogenize with 50 mL of PBS (pH 7.0) and stirred for 1 h at room temperature. The samples were filtered through filter paper and centrifuged at 8000 rpm for 8 min. Supernatant was collected and take 20 mL of supernatant for OP pesticide measurements.

OP compounds present in the sample were hydrolyzed to 4-nitrophenol by the OPH enzyme immobilized onto PVC surface. Optimum potential was applied to oxidize 4-nitrophenol and the response current generated was directly proportional to the OP compound concentration in the sample. This electrochemical technique is employed to determine OP pesticide concentration in food and water samples.

## 3. Results and Discussion

### 3.1. Purification of Organophosphorus Hydrolase from* Brevundimonas diminuta*

Enzyme was purified from crude extract by gel filtration on Sephadex G-100 and ion exchange chromatography on DEAE-sepharose. The enzyme was purified by 14.15-fold to a specific activity of 20.81 U/mg of protein from the crude OPH solution with a yield of 19.92%. The purified enzyme showed a single band at 36 KDa on SDS-PAGE.

### 3.2. Covalent Immobilization of OPH onto PVC Surface

Covalent immobilization of enzyme on PVC beaker surface was carried out by method described previously [29]. Inner surface of PVC beaker (30 mL) capacity was treated with 2 mL of fuming nitric acid at 30°C for about 2 h. After that PVC beaker was washed first with running tap water and then with double distilled water. The surface was then treated with 1 mL of methyl cyanide for 1 h and thereafter with concentrated HCl (1 mL) for 2 h. The activated surface of PVC was rinsed with distilled water and incubated with 1.25% (w/v) glutaraldehyde for about 8 h at 30°C. The surface was then washed with distilled water to remove unbound glutaraldehyde. Finally, PVC beaker was poured with 5 mL of enzyme solution containing 0.245 mg of OPH and kept overnight at 4°C. Then the surface was washed with buffer to remove the unbound enzyme.

### 3.3. Scanning Electron Microscopy of PVC Surface

**Figure 3:**
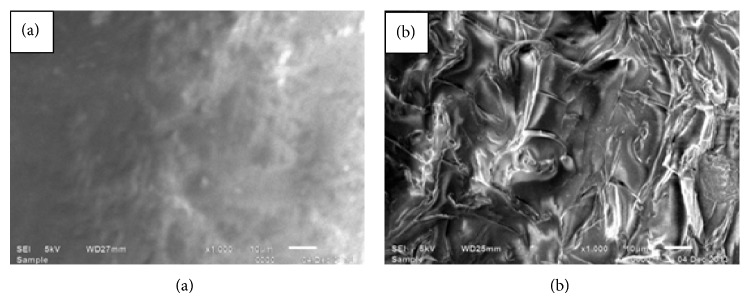
Scanning electron micrographs of a chemically modified PVC surface without (a) and with (b) immobilized enzyme.

The morphology of PVC beaker surface before and after the immobilization of enzyme was studied by SEM analysis. The change on the chemically activated PVC surface (Figure 3) was observed after immobilization method. Such a change in surface morphology of the PVC surface after immobilization method confirms the enzyme immobilization.

### 3.4. Determination of Working Potential of the OPH Biosensor

**Figure 4:**
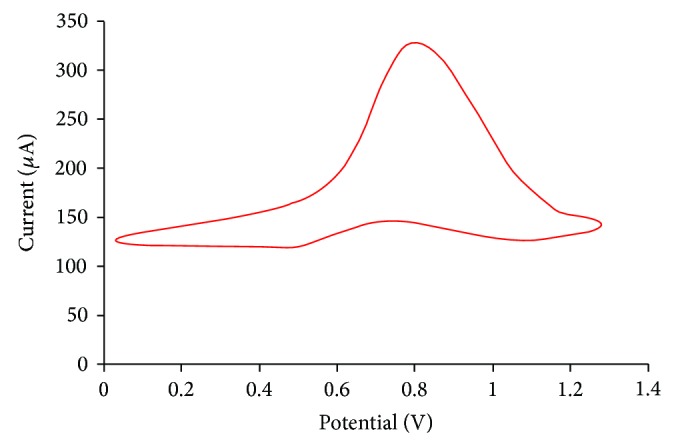
Working electrode showing oxidation peak.

The amperometric determination of OP pesticides by OPH-based biosensor depends on the anodic detection of 4-nitrophenol (enzymatically liberated product) by the electrode [19]. The working electrode showed a well-defined oxidation peak at a potential of +0.8 V (Figure 4). Therefore, +0.8 V was selected as the working potential for the further studies.

### 3.5. Kinetic Study of the OPH Biosensor

#### 3.5.1. Effect of pH

**Figure 5:**
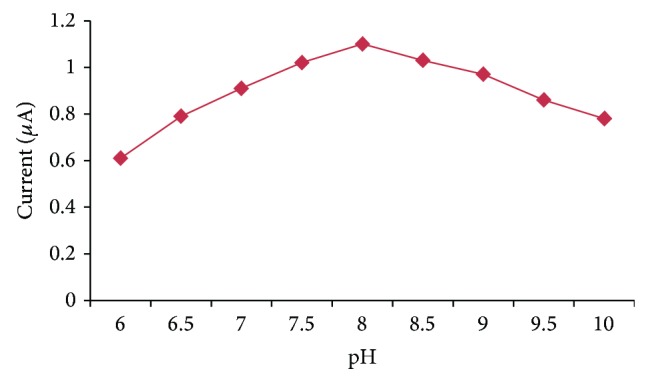
Effect of pH on the response of OPH biosensor.

The effect of pH on the biosensor response in 0.1 M succinate buffer (pH 6.0, 6.5, and 7.0), sodium phosphate buffer (7.5, 8.0, and 8.5), and borate buffer (pH 9.0, 9.5, and 10.0) in 6.0–10.0 range was studied. The optimum pH for present method was 8.0 (Figure 5) which is higher than pH-7.4 [19, 23, 30, 31], pH 7.0 [32, 33], and lower than pH 8.8 [34] and pH 9.5 [17]. This pH is similar to those reported in previous study [35].

#### 3.5.2. Effect of Incubation Temperature

**Figure 6:**
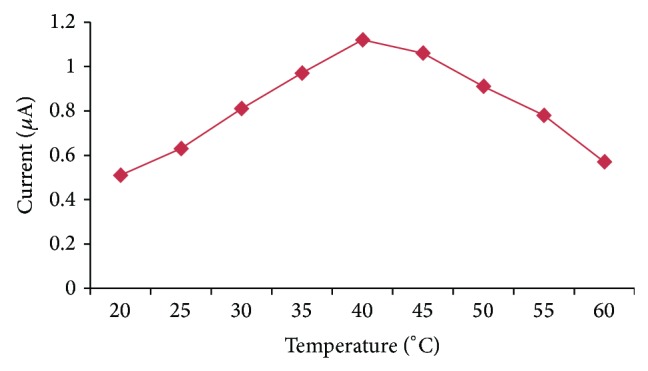
Effect of incubation temperature on the response of OPH biosensor.

The effect of the temperature on the biosensor response from 20°C to 60°C at a regular increase of 5°C was also investigated. The optimum temperature for present method was 40°C (Figure 6). The microenvironment provided by support used for immobilization makes it thermally stable and maintains its biological activity.

#### 3.5.3. Effect of Time of Incubation

**Figure 7:**
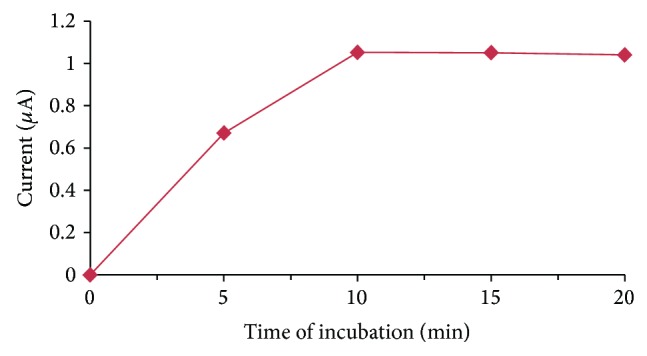
Effect of incubation time on the response of OPH biosensor.

Time of incubation was also studied from 5 min to 20 min at a regular increase of 5 min. The response of the OPH biosensor was increased up to 10 min after which no increase in response was observed. The optimum incubation time for present method was 10 min (Figure 7).

#### 3.5.4. Effect of Substrate Concentration

**Figure 8:**
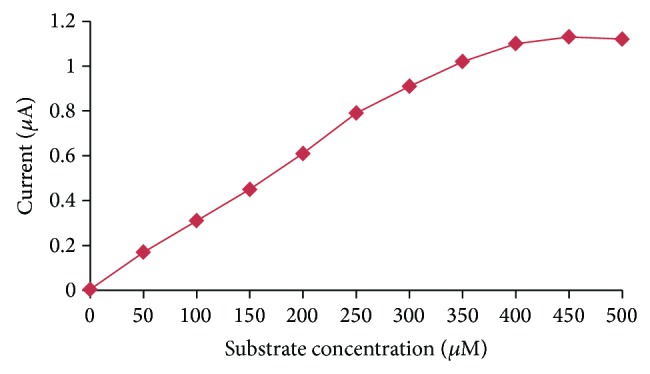
Effect of methyl parathion concentration on the present method.

The effect of methyl parathion concentration on the response of present biosensor was studied up to 500 *μ*M at an interval of 50 *μ*M. The present method showed a hyperbolic relationship between its response and methyl parathion concentration up to a final concentration of 400 *μ*M (Figure 8) after which no significant improvement in response was observed. Up to a concentration of 400 *μ*M, the OPH biosensor showed a good response to methyl parathion.

#### 3.5.5. *K*_*m*_ and *I*_max⁡_

**Figure 9:**
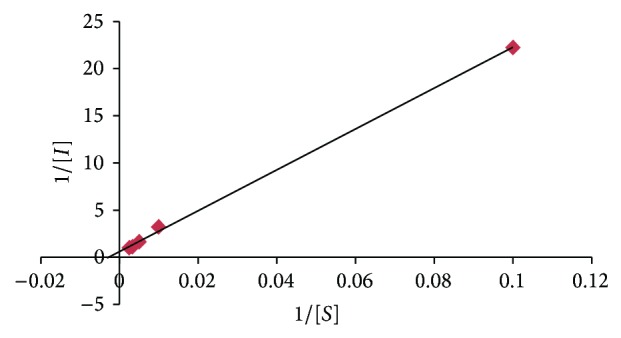
Lineweaver-Burk plot of the present method.

*K*_*m*_ and *I*_max⁡_ were determined by calculating the slope and intercept for the reciprocal plot of current versus methyl parathion concentrations, that is, Lineweaver-Burk plot. Lineweaver-Burk plot between the reciprocals of methyl parathion concentration and response current for biosensor was linear. *K*_*m*_ and *I*_max⁡_ were 322.58 *μ*M and 1.1 *μ*A (Figure 9).

### 3.6. Evaluation of the Biosensor

#### 3.6.1. Linear Working Range and Minimum Detection Limit

The linear working range and minimum detection limit of the present biosensor were calculated from standard graph between response current and substrate concentration (methyl parathion). The linear range for methyl parathion was found to be 0.1–200 *μ*M.

#### 3.6.2. Analytical Recovery

The reliability of the present method was determined by the analytical recovery of added methyl parathion (Table 1). The mean analytical recovery of added methyl parathion (5 *μ*M and 10 *μ*M) was 98.6% and 99.1%, respectively.

#### 3.6.3. Precision

To study reproducibility of the present method, the OP pesticide level was determined in the sample repeatedly on the same day (within batch) and in the same sample after storage at 4°C for one week (between batch). The results of within batch and between batch coefficients of variation (CVs) were <1.58% and <1.78% (Table 2).

#### 3.6.4. Accuracy

**Figure 10:**
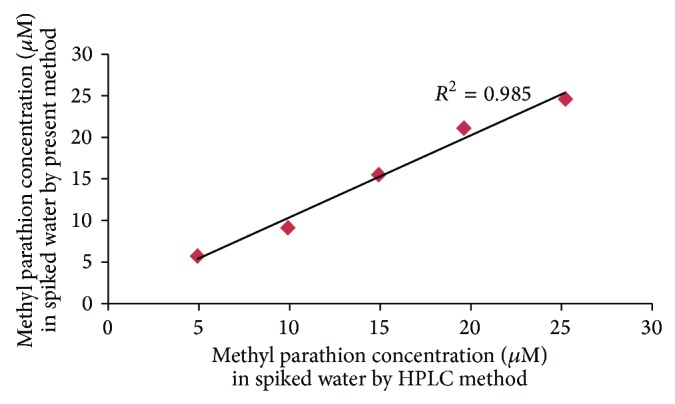
Correlation between values of methyl parathion spiked water samples determined by standard HPLC method (*x*-axis) and by the present method (*y*-axis).

To study the accuracy of the present method, the levels of methyl parathion in 5 spiked water samples were determined by the standard method (*x*) and by the present method (*y*) (Figure 10). The result obtained by the present method was in agreement with the standard method with a correlation of 0.985.

### 3.7. Reusability and Storage Stability of OPH Biosensor

**Figure 11:**
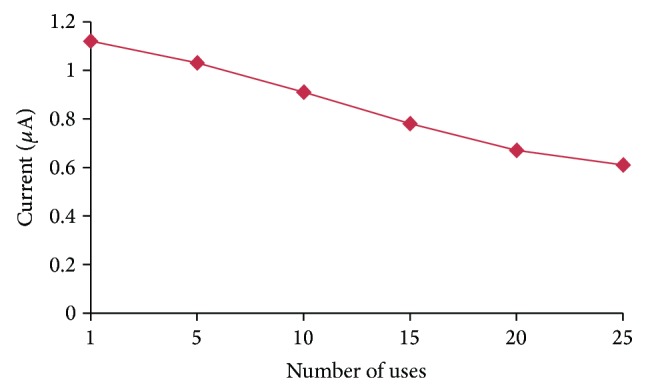
Reusability of the OPH biosensor.

**Figure 12:**
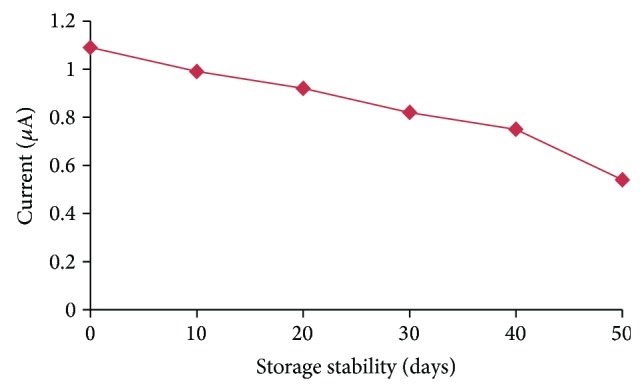
Storage stability of the OPH biosensor.

The present biosensor lost 50% of its initial activity after its 25 uses over a period of 50 days (Figures 11 and 12), when stored at 4°C, which is better than previously reported biosensors [17, 33].

### 3.8. Interference Study

Among the various substances investigated for possible interference on the response of the present method, none caused any significant interference (Table 3).

### 3.9. Application of Present Method in Determination of OP Pesticides in Food and Water Samples

The OP pesticides in differed samples were determined by the newly developed biosensor. OP pesticide level in fruits and vegetables was found in the range of 0.146 mg/kg–0.243 mg/kg and 0.017 mg/kg–0.031 mg/kg, respectively (Table 4). The OP pesticide content of water samples was also determined and found in the range 0.0029–0.0091 mg/lit (Table 5).

## 4. Conclusion

The use of carbon nanotubes as electrode material facilitates the enhanced amperometric determination of OP pesticides. The carbon nanotubes based transducer provides sensitive and stable detection of the 4-nitrophenol (enzymatically liberated product of OP pesticides). The use of OPH for biorecognition element and carbon nanotubes electrode for amperometric determination provides advantages over AChE-based biosensors that are less specific towards OP pesticides and requires multiple steps. PVC has emerged as an ideal support for enzyme immobilization and its use in designing of present electrochemical cell has proved it better than other reported biosensors. The present method provides a laboratory based analytical method for the determination of OP pesticides with high specificity. It provides fast determination of OP pesticides as well as in-field monitoring. The present method can act as a model for the development of indigenous OP pesticide sensor in miniature form.

## Figures and Tables

**Table 1:** Analytical recovery of added methyl parathion in sample.

Methyl parathion added (*μ*M)	Methyl parathion found (*μ*M) mean (*n* = 6) ± SD	% recovery
5	4.93 ± 0.02	98.6
10	9.91 ± 0.02	99.1

**Table 2:** Within and between assay coefficients of variation for determination of OP pesticide in the samples by using the present method.

*n*	OP pesticide (*μ*M) mean ± SD	CV (%)
Within batch (6)	1.84 ± 0.02	1.58
Between batch (6)	1.83 ± 0.03	1.78

**Table 3:** Effect of various interfering species on response of the present method.

Compound added (final conc. 2 *μ*M)	% relative response
None	100
Fructose	99.5
Glucose	102
Sucrose	99.4
Zn(II)	98.3
Cu(II)	98.7
Cd(II)	99.2
Ni(II)	97.3
Pb(II)	97.7

**Table 4:** OP pesticide concentration in different food samples.

Serial number	Sample	Concentration of OP pesticide (mg/kg) (*n* = 3)
1.	Grape	0.146
2.	Apple	0.243
3.	Cauliflower	0.031
4.	Cabbage	0.017

*n*: number of assays.

**Table 5:** OP pesticide concentration in different water samples.

Serial number	Sample	Concentration of OP pesticide (mg/lit) (*n* = 3)
1.	Pond water	0.0091
2.	Canal water	0.0037
3.	Bore-well water	0.0029

*n*: number of assays.
